# Difficult intubation and postoperative aspiration pneumonia associated with Moebius syndrome: a case report

**DOI:** 10.1186/s12871-022-01859-3

**Published:** 2022-10-11

**Authors:** Aya Oda, Kana Oue, Yuki Oda, Shima Taguchi, Tamayo Takahashi, Akari Mukai, Mitsuru Doi, Yoshitaka Shimizu, Masahiro Irifune, Mitsuhiro Yoshida

**Affiliations:** 1 Department of Dental Anesthesiology, Division of Oral and Maxillofacial Surgery and Oral Medicine, Hiroshima University Hospital, 1-2-3 Kasumi, Minami-ku, Hiroshima, Hiroshima 734-8551 Japan; 2 Department of Special Care Dentistry, Hiroshima University Hospital, 1-2-3 Kasumi, Minami-ku, Hiroshima, Hiroshima 734-8551 Japan; 3 Department of Anesthesiology and Critical Care, Hiroshima University, Hiroshima, Japan; 4 Department of Dental Anesthesiology, Graduate School of Biomedical and Health Sciences, Hiroshima University, Hiroshima, Japan

**Keywords:** Moebius syndrome, Postoperative pulmonary complication, Oral abnormality, Dysphagia, Cerebral palsy, Difficult airway

## Abstract

**Background:**

Moebius syndrome is a rare congenital disorder characterized by non-progressive palsy of the abducens (VI) and facial (VII) cranial nerves. Its common features include dysfunctions associated with other cranial nerves, orofacial abnormalities, skeletal muscle hypotonia, and other systemic disorders of differing severities. There are several concerns in the perioperative management of patients with Moebius syndrome.

**Case presentation:**

We present a report on the management of general anesthesia of a 14-year-old male patient with Moebius syndrome who was scheduled for mandibular cystectomy. The patient was diagnosed with Moebius syndrome at the age of 7 years based on his clinical manifestations of nerve palsy since birth and cranial nerve palsy of the trigeminal (V), facial (VII), glossopharyngeal (IX), vagus (X), and sublingual nerves (XII). The patient’s oral morphological abnormalities made intubation difficult. He also experienced dysphagia and aspiration pneumonia on a daily basis. Oral secretions were frequently suctioned postoperatively. However, after discharge, the patient developed aspiration pneumonia and was readmitted to the hospital.

**Conclusions:**

The main problem arising when administering general anesthesia to patients with this syndrome is difficult airway management. The oral abnormalities in these patients, such as small jaw and extreme dental stenosis, make mask ventilation and intubation difficult. Furthermore, this syndrome often involves respiratory impairment and dysphagia due to cerebral nerve palsy, so there is a high risk of postoperative respiratory complications. Since multiple organs are affected in patients with Moebius syndrome, appropriate perioperative management strategies must be prepared for these patients.

## Background

Moebius syndrome is a rare congenital disorder characterized by non-progressive palsy of the abducens (VI) and facial (VII) cranial nerves. In 1888 and 1892, Moebius summarized previously reported cases of congenital bilateral facial and abducens nerve palsy [1, 2]. The common features of this syndrome are dysfunctions associated with cranial nerves impairment, orofacial abnormalities, and skeletal muscle hypotonia, resulting in a variety of symptoms [3]. Patients with Moebius syndrome often have a wide range of associated malformations, including other cranial nerve defects (III, IV, V, IX, X, XII), abnormalities of maxillofacial morphology (e.g., hypoplastic tongue, small jaw, cleft palate) and limb morphology (e.g., various degrees of limb loss, entropion, finger hypoplasia), and cardiac disease (e.g., epicardial disease, septal defect). These disorders may be accompanied by respiratory impairment, muscle hypotonia, delayed language development, dysphagia, slurred speech, intellectual disability, or impaired coordination. Most cases have been isolated; however, a few familial cases have been reported [4]. The frequency of Moebius syndrome is approximately 2–20 cases per million live births [5–7]. The cause is thought to be fetal ischemia (watershed infarction) [8] due to abnormal fetal positioning, exposure to teratogens (benzodiazepines, misoprostol, cocaine, alcohol, hyperthermia, hypoxia, and rubella) during the first trimester of pregnancy [9], or developmental disorders of the brainstem (rhombencephalon) due to genetic factors [10]. Perioperative airway management in Moebius syndrome patients can be difficult owing to morphological and functional abnormalities of the orofacial region [11–15]. This disorder can lead to problems not only in airway management but also in perioperative anesthesia management, such as postoperative aspiration pneumonia [11, 12]. Here, we present a report on the anesthetic management of a patient with Moebius syndrome, which led to difficult intubation and the development of postoperative aspiration pneumonia. The patient’s parents provided written informed consent for the publication of this case report.

## Case presentation

A 14-year-old boy (height, 135 cm; weight, 26 kg) with Moebius syndrome was scheduled to undergo surgery to remove a mandibular cyst under general anesthesia. The mandibular cyst was a dentigerous cyst developed around an impacted canine. The patient was diagnosed with Moebius syndrome at the age of 7 years based on clinical symptoms, including facial paralysis, dysphagia, and incomplete quadriplegia since birth. Cerebral nerve palsy affected his trigeminal (V), facial (VII), glossopharyngeal (IX), vagus (X), and hypoglossal nerves (XII). It was difficult for him to walk and maintain a sitting position owing to decreased muscle strength in his extremities. He also had a severe intellectual disability; therefore, he needed assistance with all activities of daily living. He was intellectually impaired but was able to follow instructions such as making a peace sign to the camera, allowing the measurement of blood pressure, opening his mouth wide, and allowing the collection of blood samples (with physical restraint). However, he was incapable of engaging in meaningful verbal communication. He underwent gastrostomy at the age of one for lack of oral intake caused by dysphagia arising from cerebral nerve palsy. He often experienced aspiration pneumonia and was hospitalized for it at least once a year. He had a history of epilepsy but had not experienced epileptic seizures for more than 2 years at the time of admission. He was on carbamazepine, pranlukast hydrate, L-carbocisteine, cetirizine hydrochloride, and a combination of sodium/potassium/magnesium.

**Fig. 1:**
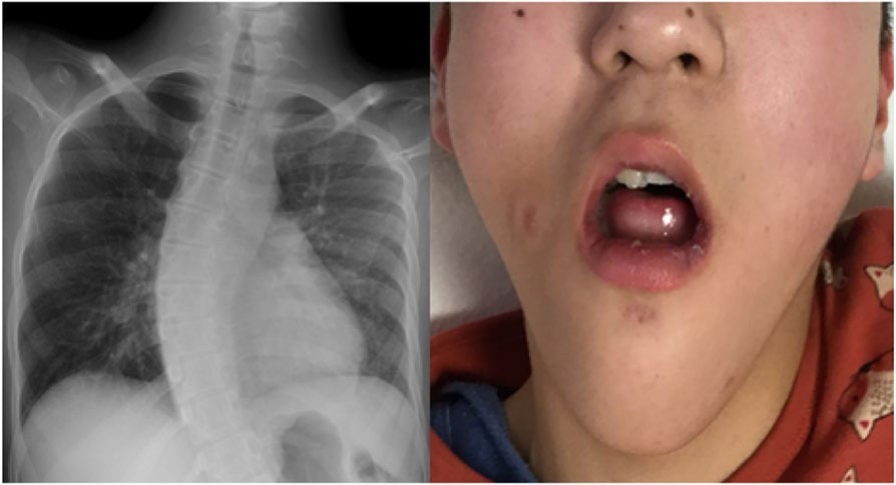
Chest radiograph and facial photograph of the patient. The left photo shows a chest radiograph of the patient. The right photo shows the patient’s facial appearance

**Fig. 2:**
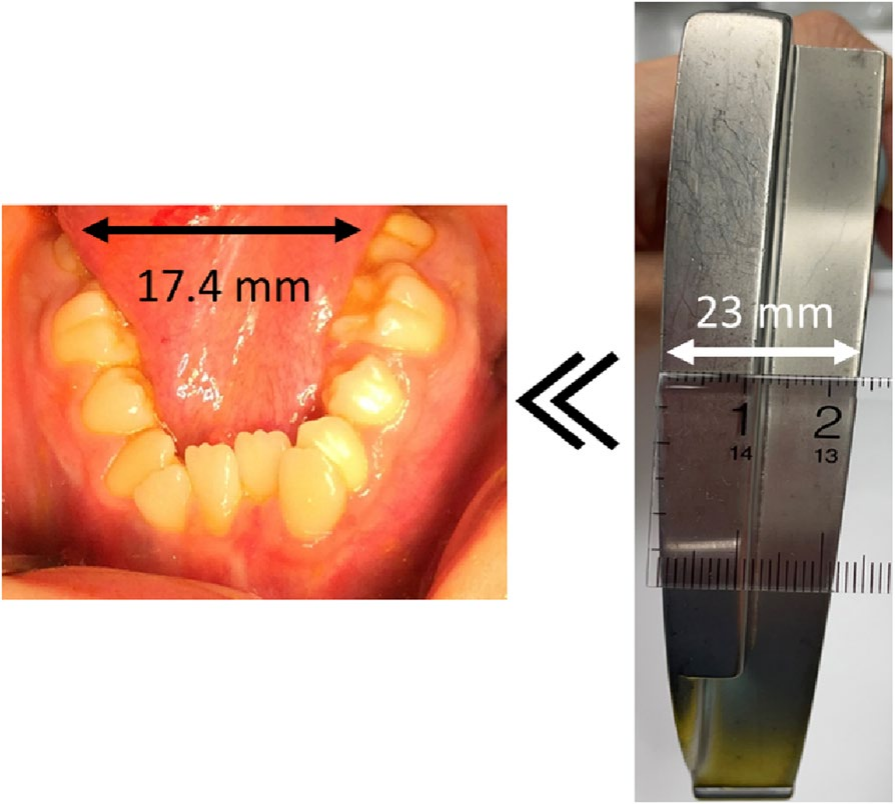
Width between the patient’s mandibular second molars compared to the Macintosh laryngoscope blade #3. The width between the patient’s bilateral mandibular second molars and the width of the Macintosh laryngoscope blade #3 were 17.4 mm and 23.0 mm, respectively. The distance between the mandibular second molars was narrower than the width of the Macintosh laryngoscope blade #3

On physical examination, we measured a blood pressure of 91/73 mmHg, a heart rate of 98/min, a respiratory rate of 14/min, an arterial oxygen saturation of 96%, and a body temperature of 35.7 **°**C. There was no heart murmur and no rhythm or waveform abnormality on his electrocardiogram. The lung fields were clear to auscultation. There were no morphological abnormalities in his extremities. He had scoliosis and oral morphological abnormalities, including extreme dental arch stenosis in both the upper and lower jaws, small-sized jaw, and a completely open bite (Fig. 1). The maximal interincisal opening was 18 mm, and the intra-arch distance between the mandibular second molars was 17.4 mm, which is narrower than the blade width of the Macintosh laryngoscope (Fig. 2). Therefore, intubation with the Macintosh laryngoscope was expected to be difficult, although the fit of the facemask was good and there were no problems with airway patency.

**Fig. 3:**
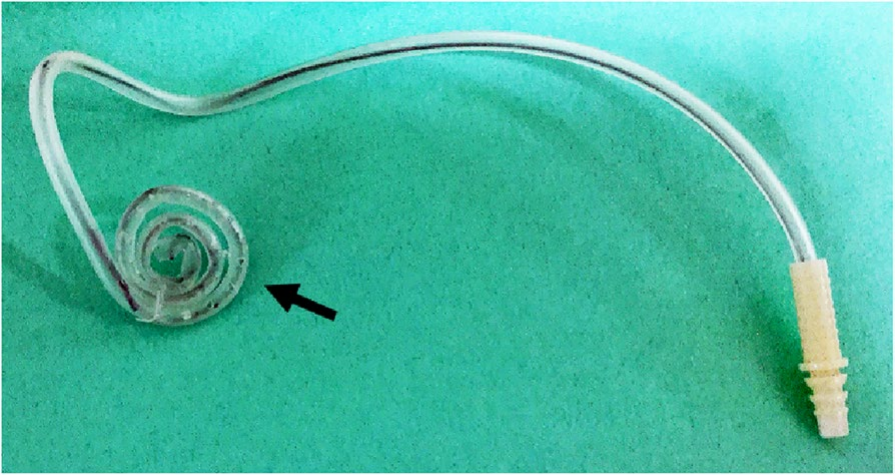
Continuous suction tube. Original suction tube prepared for continuous intraoral suction after surgery. The tube was inserted with a wire to maintain the spiral shape, and we made several holes on the side of the tube (closed arrow)

The patient had significant oral secretions on a daily basis. Therefore, to prevent aspiration after surgery, we prepared an original suction tube that could be placed in the oral cavity to aspirate oral secretions and bleeding continuously. For ease of placement in the mouth and to maintain the spiral shape of the tube, a wire was inserted into the tube, and several holes were made in it (Fig. 3).

Although he came to the operating room without any premedication, he complied with the intravenous line placement calmly with slight physical restraint. Atropine sulfate was administered to inhibit secretion, followed by rapid induction using propofol (2 mg/kg) and remifentanil (0.5 μg/kg/min). Once mask ventilation was achieved, which was an easy process, a muscle relaxant (rocuronium) 10 mg was administered.

By preoperative intraoral measurement, it was expected that the distance between the bilateral molars was too small to insert the blade of the Macintosh laryngoscope into the pharynx. Therefore, we chose a McGRATH™ MAC video laryngoscope (blade size #3; Aircraft Medical, UK) as the first option, and we also prepared a fiberoptic bronchoscope in case of difficulties. Since the bilateral molars interfered with the blade, only a Cormack–Lehane grade III view of the vocal cords was secured even with the McGRATH™ MAC device. Thus, the entire epiglottis was raised by the blade to identify the glottis. Finally, we nasally intubated the patient using a nasal 6.0 cuffed endotracheal tube (Portex® North Polar tube [Ivory PVC Portex tube; Smiths Medical International, Hythe, UK]).

General anesthesia was maintained with propofol (6–10 mg/kg/h) and remifentanil (0.3–0.6 μg/kg/min), and the depth of anesthesia was monitored with entropy (GE Datex-Ohmeda Entropy, Chicago, IL, USA). The patient’s vital signs were stable during the operation. Acetaminophen (400 mg) was administered intravenously for postoperative analgesia, and the scheduled treatment was completed without complications. The removed cyst, about 10 mm × 10 mm in size, included the right permanent canine and a supernumerary tooth. Before extubation, we confirmed recovery from the effect of the muscle relaxant using the NeuroMuscular Transmission monitor (GE Healthcare, Chicago, IL, USA) and confirmed reliable hemostasis along with sufficient intraoral suction. There was an excessive amount of oral secretion. Once the recovery of spontaneous respiration, body movement, spontaneous eye-opening, and deglutition reflex were confirmed, the patient was extubated.

After extubation, he was conscious, calm, and not excitable; he did not cry but refused to wear an oxygen mask. Thirty-five minutes of observation in the operation room was followed by prompt transfer to the ward for proper monitoring. As he was not somnolent in the ward, it was difficult to place the continuous suction tube (Fig. 3); therefore, the nurse and mother performed frequent oral suctioning. When secretions accumulated in the oral cavity, the patient used the suction tube by himself. He left the hospital the next day in good general condition. A five-day supply of antibiotics (amoxicillin, 375 mg/day) was prescribed to prevent wound infection. However, he developed a fever (temperature, 38.5 **°**C) on postoperative day 5 and was diagnosed with aspiration pneumonia. He was treated with intravenous infusion antibiotics and hospitalized at another center for a week.

## Discussion and conclusions

Moebius syndrome is a rare congenital disorder characterized by non-progressive palsy of the abducens (VI) and facial (VII) cranial nerves. The various symptoms and pathologies associated with Moebius syndrome lead to a variety of problems in perioperative management. To date, approximately 800 reports of Moebius syndrome have been published, of which approximately 20 have been related to anesthesia (and approximately 10 to general anesthesia).

**Fig. 4:**
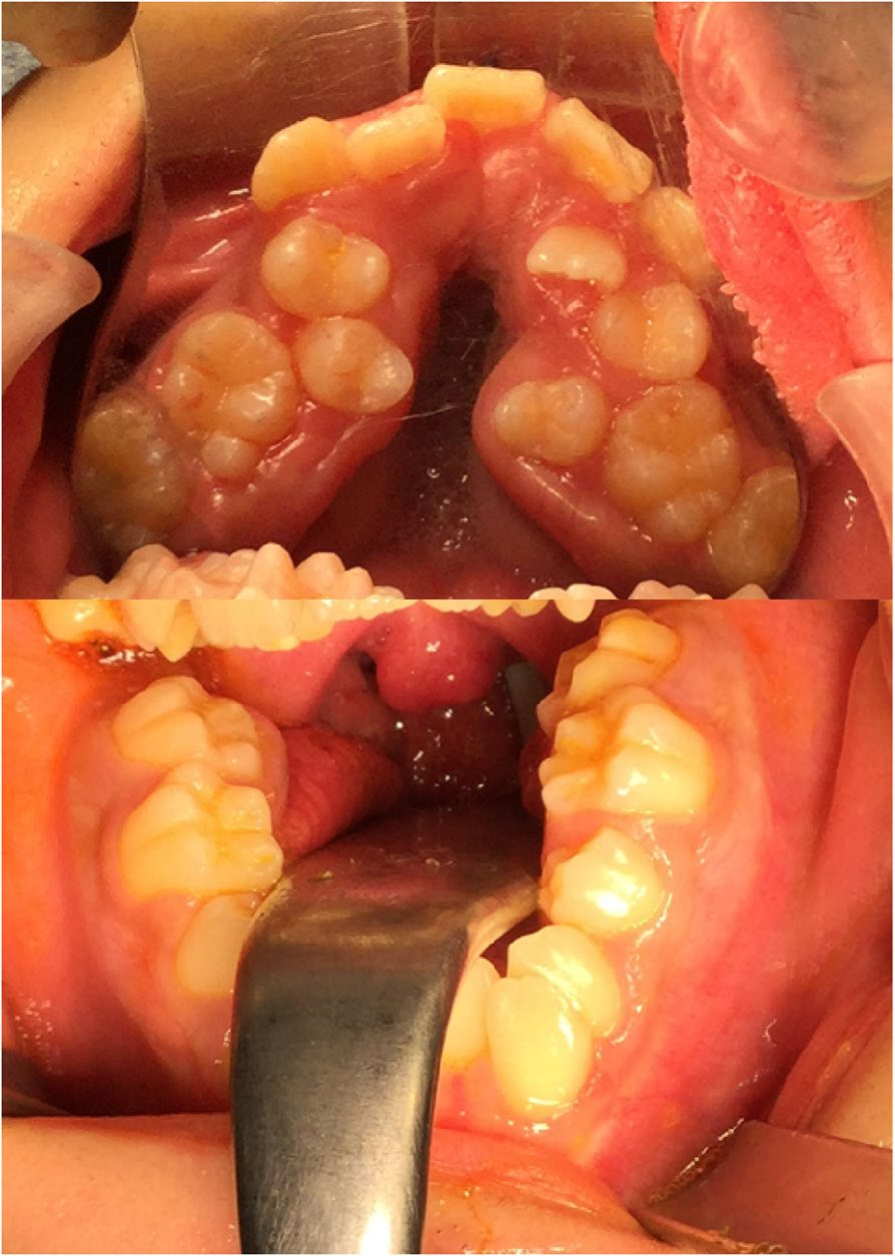
Intraoral view. The patient had oral morphological abnormalities such as extreme dental arch stenosis in both the upper and lower jaws

We faced some limitations during the perioperative anesthesia management of this patient. The first problem was airway management. The oral abnormalities, such as a small jaw or extreme dental stenosis, can cause difficulties in mask ventilation and intubation. There are several reports on airway management of patients with Moebius syndrome [11–15], and mask ventilation has often been reported to be easy [13]. We estimated that mask ventilation was available based on his physical findings. Therefore, in this case, we planned to perform rapid induction with propofol and remifentanil followed by tracheal intubation. As a matter of fact, the mask fit was good, and mask ventilation was achieved easily. After induction of anesthesia, we administered a muscle relaxant to prevent noxious reflexes during intubation. However, there is a risk of airway loss in this syndrome due to tracheomalacia and palatal and uvular weakness. There have been reports of cardiac arrest after emergency tracheostomy due to airway obstruction upon induction of general anesthesia in this syndrome [12]. Although it was not possible due to lack of patient cooperation, we believe it would have been safer to perform awake fiberoptic intubation or to secure the airway with spontaneous breathing remaining. Previous reports have found that overweight and obesity [15], abnormalities of mandibular/palatal structures, and four cranial nerves (IX, X, XI, XII) are statistically significant factors associated with tracheal intubation difficulties in patients with Moebius syndrome [13]. Although our patient was not overweight, several factors made tracheal intubation difficult, including mandibular/palatal abnormalities (Fig. 4) and cranial nerve palsy (IX, X, and XII). In addition, the distance between the mandibular second molars (17.4 mm), measured using calipers before the surgery, was narrower than the blade size of the Macintosh laryngoscope (23.0 mm wide) (Fig. 2), predicting that intubation with the Macintosh laryngoscope would be difficult. Therefore, we planned to use a McGRATH™ MAC video laryngoscope. While it was difficult to perform laryngeal deployment as usual, intubation was achieved with the McGRATH™ MAC.

The second problem was related to the risk of postoperative pulmonary complications. This syndrome often involves respiratory impairment and dysphagia that can increase the risk of postoperative respiratory complications due to regurgitation and aspiration of oral secretions or gastric contents during the perioperative period [11]. In general, cranial nerves play an important role in regulating all the phases of swallowing [16]. Therefore, impairment of the cranial nerves (especially V, VII, IX, X, and XII) can lead to decreased laryngeal behavior, pharyngeal retention after swallowing, decreased vocal fold adduction leading to dysphonia and decreased coughing effectiveness, and silent aspiration [17]. In our patient’s case, dysphagia was observed because cerebral nerves IX, X, and XII were affected. In addition, silent aspiration is not related to food intake and often occurs when aspiration worsens owing to a decrease in the cough reflex at night [18]. Therefore, even patients who have undergone gastrostomy and have no oral intake should be carefully monitored [19]. Although our patient had no oral food intake in the perioperative period, he was hospitalized for aspiration pneumonia at least once a year; therefore, it was highly probable that he aspirated oral secretions on a routine basis due to dysphagia. Hence, the patient was at a particularly high risk of aspiration pneumonia in the perioperative period. Therefore, anesthesia was planned to prevent aspiration as much as possible. First, no premedication was administered to avoid functional impairment of swallowing and cough reflexes due to somnolence. Although we were concerned about the increase in secretions due to weeping, we thought it would be possible for him to enter the operating room and receive an intravenous line without premedication, considering his cooperation during blood collection and in general. In addition, an anticholinergic agent, atropine sulfate, was administered immediately after securing the venous route to control oral secretions [11, 14]. Anticholinergic drugs can also cause depression of cough reflex, impairment of ciliary motility, and increased risk of postoperative pulmonary complications. However, in studies of Moebius syndrome, there were significant problems of partial airway obstruction, pulmonary aspiration, and hypoxemia due to secretions during recovery, so the use of antisialagogues could be recommended [11, 14]. That was a difficult decision, but we used atropine sulfate because of excessive saliva production in this case.

In general anesthesia, if the cough reflex is weakened or ciliary motor function is decreased due to the effects of intraoperative anesthetics, muscle relaxants, or opioids used for postoperative pain management, the risk of postoperative pneumonia increases because of difficulty in the excretion of airway secretions [20]. Therefore, for patients with a high risk of postoperative pulmonary complications, the drugs used in the perioperative period must be carefully selected, and aspiration prevention strategies must be applied. In this case, to avoid weeping during emergence agitation and postoperative nausea/vomiting and ensure quick awakening, we did not use inhalation anesthetics but used propofol, which suppresses the pharyngeal reflex, but its effects wear off quickly [21]. In addition, because of abnormalities in ventilatory control of both central hypoventilation [22] and idiopathic tachypnea [23] in patients with Moebius syndrome owing to brainstem lesions, the use of fentanyl, which may cause respiratory depression due to residual effects, was avoided, and the ultra-short acting remifentanil was used. Furthermore, we planned to achieve reliable hemostasis and continuous postoperative intraoral suctioning because of the risk of bleeding drips and increased oral secretions due to the irritation of intubation and intraoral surgery. With these plans, the patient had good postoperative wakefulness, and oral suctioning could be performed as needed. Nevertheless, he developed aspiration pneumonia on postoperative day 5, when his antibiotic medication had ended. In addition to the decreased swallowing function caused by this syndrome, the residual effects of anesthetic agents and increased oral secretions from oral surgery increase the risk of aspiration. Furthermore, dysphagia may occur after tracheal intubation for various reasons, such as mucosal epithelial denaturation caused by contact between the tracheal intubation tube and tracheal mucosa, delayed or lost induction of the swallowing reflex, damage to the vocal cord mucosa, and laryngeal edema by friction [24]. These factors may have led to the development of aspiration pneumonia in our patient despite various considerations.

We have determined recovery from muscle relaxants using neuromuscular monitoring of the adductor pollicis brevis muscle. However, in such patients, the adductor pollicis monitoring method would not accurately represent the response of the pharyngeal muscles. Therefore, we consider that we should have administered sugammadex to antagonize the effects of the muscle relaxant completely. In addition, frequent postoperative aspiration of oral secretions was performed, but anticholinergic drugs were not used. However, an increase in postoperative oral secretions was observed, and it may have been necessary to consider using some anticholinergic agents even after surgery. Several reports indicate that perioperative oral care [25, 26] and oral chlorhexidine [27, 28] are effective in preventing postoperative pneumonia. We believe these interventions should also have been considered to prevent postoperative complications after oral surgery, especially in patients with chronically impaired swallowing and airway protection reflexes, as in this case.

In addition, Moebius syndrome may be associated with congenital heart disease, and cardiac abnormalities were observed in 33% of the patients in a study by Bell et al. [3]. Furthermore, there have been reports of deaths due to malignant hyperthermia [29]. Because of the variety and severity of complications, perioperative management of patients with Moebius syndrome requires various considerations.

This report presents only a single case, and further case studies are needed to establish appropriate anesthesia management for patients with Moebius syndrome. However, the preoperative measurement of the distance between the bilateral mandibular second molars carried out in this study may be useful in predicting preoperative intubation difficulties not only in patients with Moebius syndrome but also in patients with other diseases that involve oral abnormalities.

Moebius syndrome is a highly variable syndrome characterized by abducens nerve palsy and facial paralysis. Some of its most common features are paralysis of other cranial nerves and abnormal maxillofacial morphology, which increase the risk of difficulties in airway management, aspiration, and postoperative respiratory complications. It is important to accurately assess the pathology, symptoms, and anatomical abnormalities of each patient with Moebius syndrome in the preoperative period and to develop an optimal perioperative anesthesia plan for these patients.

## Data Availability

All data related to this case report are contained within the manuscript.
